# Viral Organization of Human Proteins

**DOI:** 10.1371/journal.pone.0011796

**Published:** 2010-08-25

**Authors:** Stefan Wuchty, Geoffrey Siwo, Michael T. Ferdig

**Affiliations:** 1 National Center of Biotechnology Information, National Institutes of Health, Bethesda, Maryland, United States of America; 2 Eck Institute for Global Health, Department of Biology, University of Notre Dame, Notre Dame, Indiana, United States of America; Fondazione Telethon, Italy

## Abstract

Although maps of intracellular interactions are increasingly well characterized, little is known about large-scale maps of host-pathogen protein interactions. The investigation of host-pathogen interactions can reveal features of pathogenesis and provide a foundation for the development of drugs and disease prevention strategies. A compilation of experimentally verified interactions between HIV-1 and human proteins and a set of HIV-dependency factors (HDF) allowed insights into the topology and intricate interplay between viral and host proteins on a large scale. We found that targeted and HDF proteins appear predominantly in rich-clubs, groups of human proteins that are strongly intertwined among each other. These assemblies of proteins may serve as an infection gateway, allowing the virus to take control of the human host by reaching protein pathways and diversified cellular functions in a pronounced and focused way. Particular transcription factors and protein kinases facilitate indirect interactions between HDFs and viral proteins. Discerning the entanglement of directly targeted and indirectly interacting proteins may uncover molecular and functional sites that can provide novel perspectives on the progression of HIV infection and highlight new avenues to fight this virus.

## Introduction

The determination of webs of protein interactions [1], [2], [3], [4] and protein complexes [5], [6], [7], [8], [9] in many different single and multi-cellular organisms progresses at a fast pace, peaking in attempts to determine the human interactome in various ways [10], [11], [12], [13], [14]. Although such webs of intracellular interactions are increasingly well characterized, little is known about large-scale maps of protein interactions between cells. Therefore, the investigation of host-pathogen interactions is a crucial step toward a thorough understanding of an organism's pathogenesis, providing an essential foundation for the development of effective therapeutic and prevention strategies to combat diseases. Uetz et al. released the first small map of computationally inferred physical protein interactions between the human host and the Kaposi-Sarcoma associated Herpesvirus (KSHV) as well as the Varicella Zoster-Virus (VZV) [15]. In a different approach, Calderwood et al. [16] experimentally determined a map of physical protein interactions between the Epstein-Barr-Virus and the human host. Recently, Bandyopadhyay et al. [17], identified subnetworks of virus-host proteins that are expressed at different stages of the HIV-infection and Dyer et al. compared experimentally known interactions of different viruses with the human host [18]. Brass et al. utilized a comprehensive large scale-screen of siRNAs to identify HIV dependency factor proteins (HDF). Although these proteins do not directly interact with viral proteins, they play an indirect, yet important role in the infection process of HIV [19].

Here, we pooled experimentally verified interactions between HIV-1 and human proteins, along with a set of HIV-dependency factor proteins (HDF), to investigate the topology of interactions between viral and host proteins on a large scale. We found that targeted and HDF proteins appeared predominantly in rich-clubs, allowing the virus to take control of the human host by reaching protein pathways and diversified cellular functions in a pronounced and focused way. Although HIV-1 does not physically interact with HDFs, we observed that prominent transcription factors and protein kinases establish indirect links between such host and viral proteins, suggesting molecular and functional sites that can be used to systematically hamper the virus.

## Results

### Rich-clubs of proteins as viral targets

**Figure 1:**
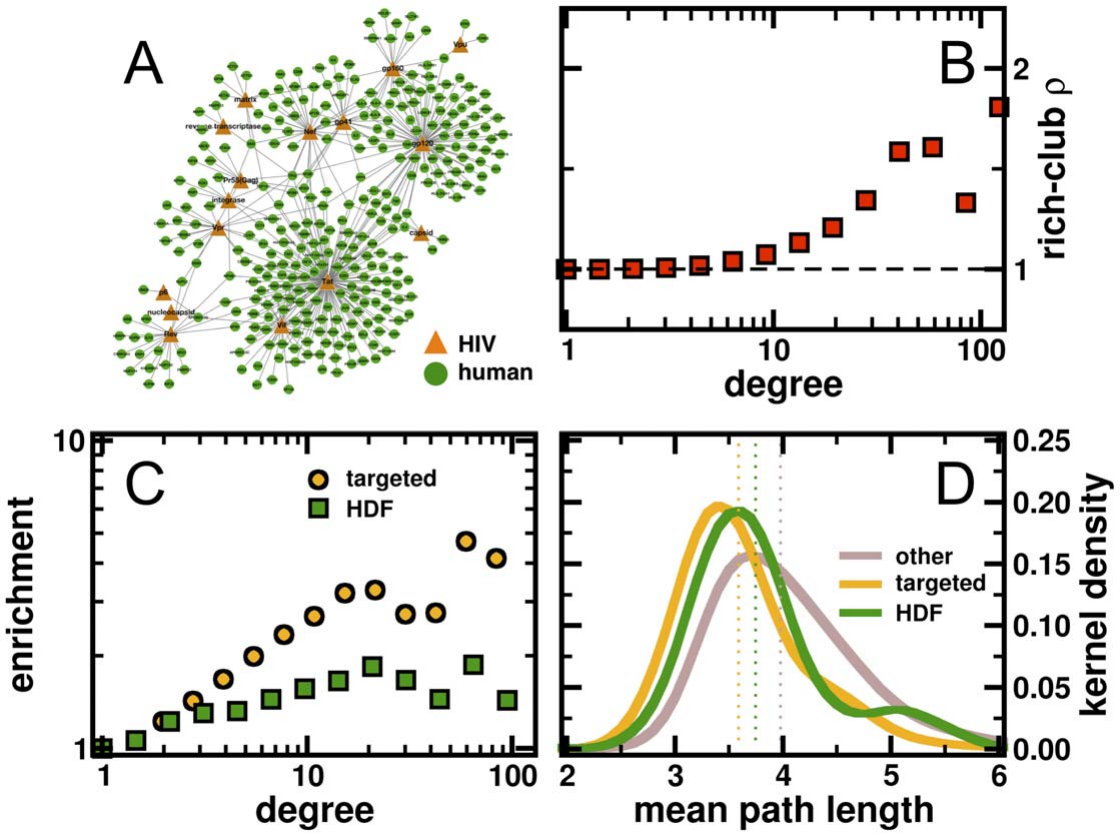
Statistics of targeted and HDF proteins. (**A**) Constructing a bipartite network of interactions between HIV-1 and human proteins, we find one connected subnetwork. Randomizing human proteins, we found that the presence of one connected network is statistically significant (P<10^−4^). (**B**) The mean rich-club coefficient ρ reflects the degree to which proteins with at least a certain number of interaction partners are intertwined among each other. We observed that ρ significantly increased with higher degrees in the human protein interaction network, indicating the presence of an oligarchy (*i.e.* rich clubs) of highly interacting and intertwined proteins. In (**C**) we determined the enrichment of targeted human host proteins in such rich clubs. Specifically, highly connected proteins appeared to be increasingly targeted by the virus. Although weaker HIV dependency factor proteins (HDF) were enriched in rich clubs as well, a difference that is statistically significant compared to the enrichment of targeted proteins (Kolmogorov-Smirnov test, P<0.01). (**D**) Utilizing a network of interactions between proteins of the human host, we determined the lengths of shortest paths for each pair of human proteins. Calculating the mean of the shortest path lengths, we found that proteins, which are targeted by the virus, have lowest means (dotted lines). Focusing on HDFs we observed a shift toward longer mean path lengths, a difference that is statistically significant compared to targeted proteins (Student's t-test, P<0.02). This observation is reinforced if we account for all remaining human proteins, results that are significantly different in comparison to targeted and HDF proteins, respectively (P<0.05).

Here, we utilized a compilation of 702 experimentally verified physical protein interactions between 17 HIV-1 and 519 human proteins. In addition, we accounted for 290 HIV dependency factor proteins (HDF) that play a role in the viral infection process [19]. Considering a graphical depiction of the web of all host-viral interactions in Fig. 1A, we observed a single connected subnetwork. Randomizing the human interaction partners of viral proteins, we found that the presence of one connected web is statistically significant (P<10^−4^). In Fig. 1A, we also observed that Tat, Nef and Vpr – viral proteins that predominantly interfere with regulatory host processes – appear to be viral hubs that interfere with the human host interactome on a combinatorial basis. As such, we will show topological features of the human interactome that are potential direct and indirect targets of HIV-1.

In contrast to other protein interaction networks of eukaryotic organisms, such as *S. cerevisiae*, *C. elegans* and *D. melanogaster* [20], [21], the human interactome is composed of an oligarchy of highly interacting and intertwined nodes. Such a rich-club phenomenon is quantified by the fraction of edges among nodes that have at least a certain number of neighbor's *k* in the actual and randomized networks. As such, the rich-club coefficient 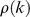 points to the presence of a core of highly intertwined nodes with degree of at least *k* if 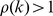. In the absence of this phenomenon (i.e. 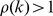) networks are dominated by many well defined functional communities which are sparsely connected by highly interacting proteins [20]. Collecting pairwise protein interactions in *H. sapiens* from public databases and accounting for phosphorylation events between kinases and other proteins, we assembled a network of 23,752 interactions between 4,075 human proteins that are expressed in the human host cell. In this network, we found a strong rich-club signal among proteins with increasing degree (Fig. 1B). Assuming that such a proteomic feature might be an exploitable target of the virus, we hypothesized that proteins in rich clubs are preferably targeted by the pathogen. In Fig. 1C, we found that there exists an enrichment of targeted proteins in rich clubs. Although weaker, yet significantly different compared to targeted proteins (Kolmogorov-Smirnov test, P<0.01), we observed that HIV dependency factor (HDF) proteins are enriched in rich clubs as well. These observations suggest that samples of highly connected and intertwined proteins provide topological features which the virus utilizes as a gateway to seize control of the host cell in a direct and indirect way. As another parameter of centrality, we calculated the mean length of shortest paths from each protein. Focusing on directly targeted host proteins, we found a bell shaped curve (Fig. 1D) at relatively short path lengths. Focusing on HDFs we observed a significant shift toward longer mean path lengths compared to targeted proteins (Student's t-test, P<0.02). If we account for all remaining human proteins, we found this trend reinforced, a result that is significantly different in comparison to targeted and HDF proteins, respectively (P<0.05).

### Viral aspects of pathways

**Figure 2:**
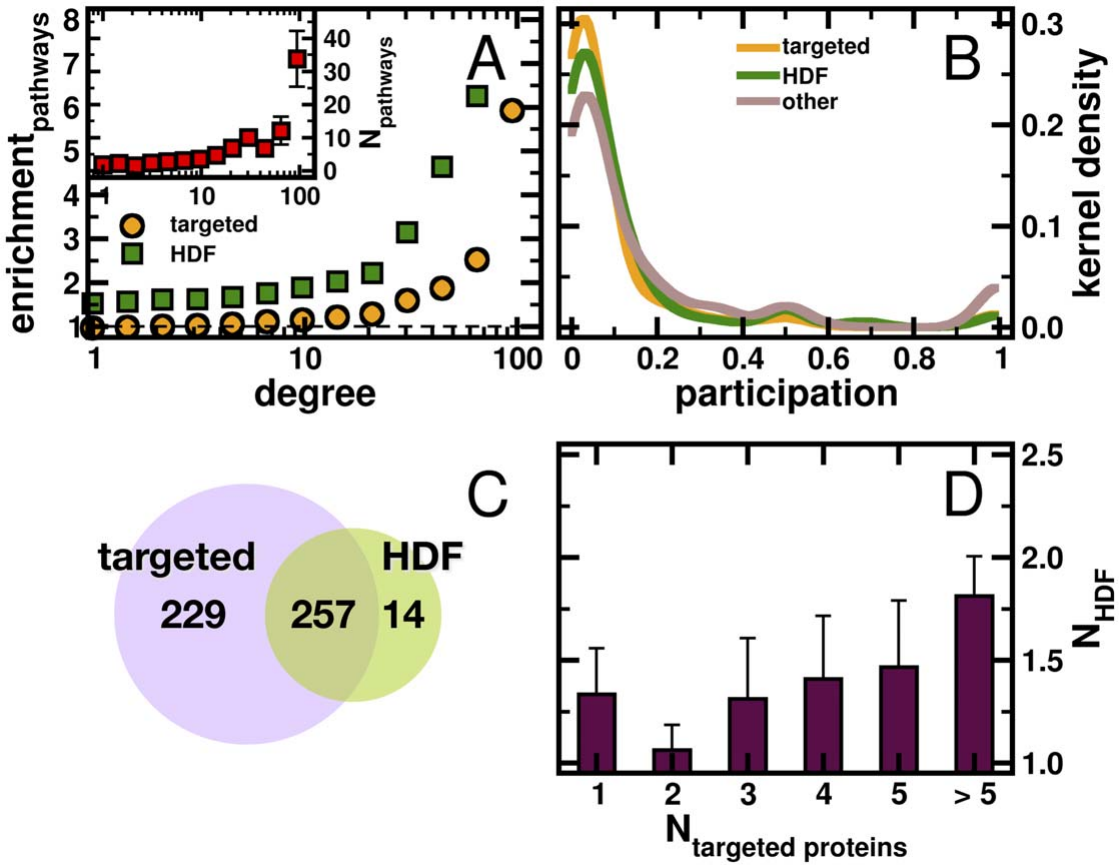
Pathway related characteristics of targeted and HDF proteins. The inset of (**A**) suggests that proteins in rich clubs tend to participate in an elevated number of pathways. Considering proteins that are targeted by HIV, we calculated the total number of pathways such proteins are involved in. In rich clubs, we found a trend toward strong enrichment of pathways that harbor targeted proteins in rich clubs of proteins. Analogously, we observed a similar trend with HIV dependent factor proteins (HDF) that was significantly different from targeted proteins (Kolmogorov-Smirnov test, P<0.005). (**B**) A low value of the pathway participation coefficient indicates that the interactions of a protein reach many different pathways and *vice versa*. Considering all targeted proteins, we obtained a maximum around low values and recovered a similar result, when we focused on all HDFs. While the difference of these distributions is insignificant (Kolmogorov-Smirnov test, P<0.3), the trend appears diminished if we consider all other human host proteins. In comparison to targeted and HDF proteins, respectively, differences are significant (P<0.01), indicating that the placement of targeted and HDF proteins buffers differences in the proteins abilities to reach into many pathways. (**C**) Counting the number of pathways that have at least one protein that is targeted by the virus we found 486, while 271 pathways had at least one HDF. Indicating a significant overlap, 257 pathways involved both targeted and HDF proteins (hypergeometric test, P<10^−45^). (**D**) In these pathways, we determined the corresponding numbers of HDFs and targeted proteins. We found a significant upwarding trend, indicating that pathways that are increasingly targeted by the virus also harbor HDFs (Pearson's r = 0.2, P<0.01).

Protein pathways are another level of systems information in which to recognize patterns that reveal how the virus exploits the host cell. This approach relies on the strength of 913 manually curated pathways from the Pathway Interaction Database [22]. Specifically, we tested whether pathways are significantly enriched for genes that are expressed in the human host cell. Applying a Fisher exact test, we found 851 enriched pathways (P<0.05, Table S1). Utilizing this set of pathways, we observed that hubs appeared in an increasing number of pathways (inset, Fig. 2A). This observation emphasizes a role of protein hubs being involved in numerous protein pathways and suggests that the pathogens have taken advantage of the host network at the pathway level. Indeed, we found that host proteins that are targeted by the virus appeared in an increasing number of pathways with increasing degree (Fig. 2A). Focusing on HDF proteins, we recovered a similar, yet significantly different trend compared to targeted proteins (Kolmogorov-Smirnov test, P<0.005).

A corollary of this result is that the comparably small number of targeted proteins and HDFs would allow the virus to interact with a larger number of pathways than would appear by chance. Out of 851 enriched pathways, all human proteins targeted by the virus were part of 486 pathways, while HDFs touched 271 pathways. Randomizing the sets of targeted and HDF proteins, we found that such numbers are smaller than expected by chance alone (P<10^−4^).

We further hypothesized that the virus tendency to target host pathway hubs effectively mediates the infection while ensuring variety, such that the virus targets numerous distinct pathways with a comparably low number of targeted proteins. As a measure of diversity, we defined the pathway participation coefficient: if a protein predominantly interacts with partners that are members of the same pathway, this measure tends toward 1, while the opposite holds if the interaction partners of the considered protein are distributed among many different pathways. Accounting for all human proteins that are neither targeted nor HDFs, we observed that interactions of a single protein occur in a variety of pathways, as indicated by the maximum around low values of the pathway participation coefficient. In turn, relatively few interactions are confined to a small number of pathways (Fig. 2B). Comparing to the subsets of human proteins that are targeted by the virus, we found a significant reinforcement of the initial diversity signal (Kolmogorov-Smirnov test, P<0.01). Similarly, for HDFs we found this signal significantly reinforced (P<0.01) as well, confirming that the use of a small subset of host proteins effectively secures a pathogen's reach into a breadth of cellular activities without inundating any particular one. However, we found no significant differences between the corresponding distributions of targeted and HDF proteins, indicating that the placement of targeted and HDF proteins in the network is defined by a cohesive pathway-dependent combination of targeted and HDF proteins. Consequently, we searched for a correlation between the number of targeted and HDF proteins in pathways. Counting the number of pathways that have at least one protein that is targeted by the virus we found 486. In turn, we found 271 pathways that harbored at least one HDF. In the Venn diagram in Fig. 2C, we observed a significant overlap where 257 pathways involved both targeted and HDF proteins (hypergeometric test, P<10^−45^). In these pathways, we found a significantly upwarding trend between the number of targeted and HDF proteins (r = 0.2, P<0.01), confirming our hypothesis that pathways which harbor targeted proteins also significantly involve HDFs (Fig. 2D).

### Direct and indirect host-virus interactions

**Figure 3:**
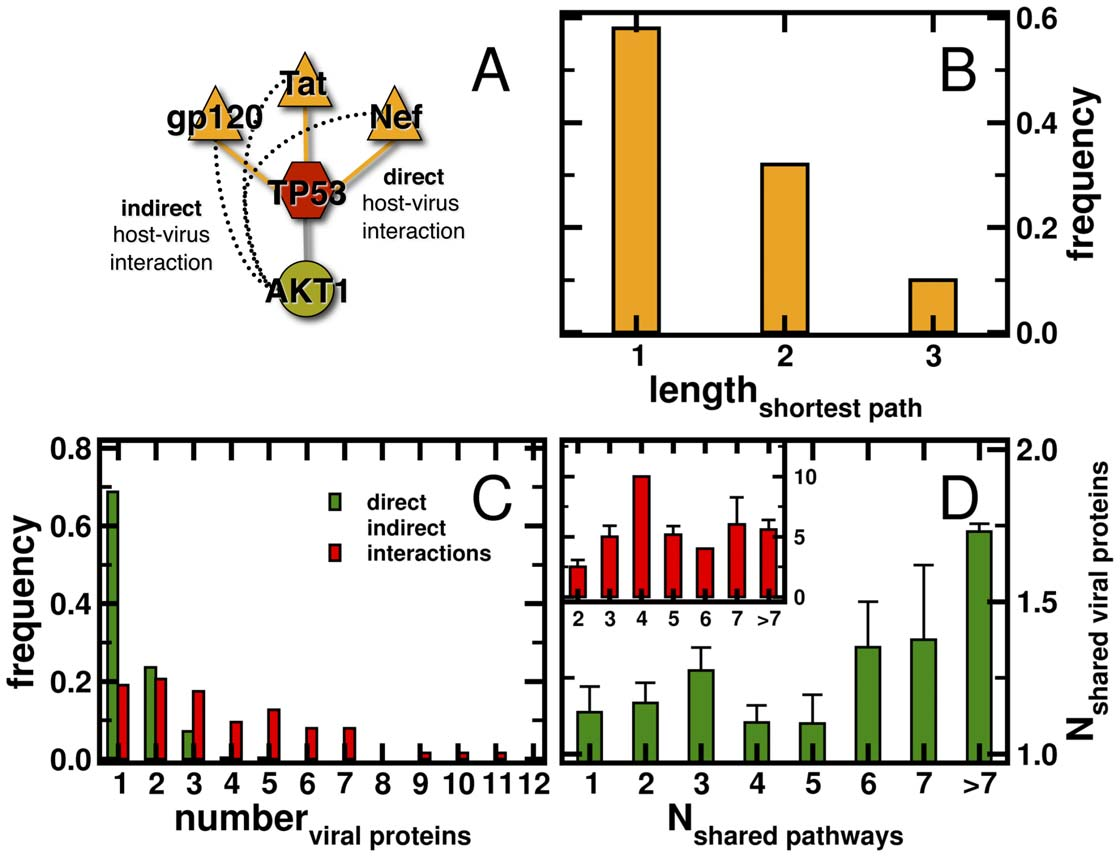
Direct and indirect host pathogen interactions. (**A**) As an example for direct host-virus interactions, we show physical interactions between viral proteins Tat, Nef and gp120 and TP53. In turn, transcription factor TP53 controls the expression of the HDF protein AKT1. Defining indirect host-virus interactions, we considered AKT1 being indirectly linked to Tat, Nef and gp120. (**B**) Calculating shortest paths from each HIV dependency factor (HDF) protein to a targeted protein, we found that the majority of HDFs are interacting with a protein that the virus attacks. (**C**) Counting the number of interacting viral proteins, we found that the majority of directly targeted host proteins binds to one viral protein. The distribution of indirect interactions as previously defined significantly shifted to higher numbers of interacting viral proteins (Student's t-test, P<10^−3^). In (**D**) we connected human proteins if they significantly co-appeared in pathways and constructed a different network, where linked proteins were significantly targeted by the same viral proteins. Comparing links in these networks, we observed a significant correlation between the number of shared viral proteins and pathways where connected host proteins co-appear in (Pearson's r = 0.47, P<0.01). Analogously, we constructed a network, linking human proteins that significantly shared indirectly interacting viral proteins where we observed a weaker correlation (inset, r = 0.26, P<0.1).

Up to this point we considered direct interactions of human host and viral proteins and regarded HIV dependency factors as proteins that are influenced by the virus in some indirect, yet unknown way. However, the integration of information about interactions between proteins can potentially help us to uncover ways viral proteins indirectly interact with HDFs through their host targets. For example, we found protein interactions between viral proteins Tat, Nef and gp120 and TP53 (Fig. 3A). In turn, transcription factor TP53 controls the expression of the HDF protein AKT1 by a protein DNA interaction. Since Tat, Nef and gp120 are connected to AKT1 through TP53, we considered AKT1 indirectly interacting with those three viral proteins. As a consequence, the length of the shortest path of AKT1 to a directly targeted host protein is 1. To determine shortest paths, we considered phosphorylation events between kinases and other proteins as directed in our host network of physical protein-protein interactions. Furthermore, we added experimentally confirmed directed protein-DNA interactions between transcription factors and proteins. Determining shortest paths between each HIV dependency factor protein to a protein that is directly targeted by virus proteins, we found that the majority of HDFs directly interacts with a targeted protein (Fig. 3B; Table S2). We counted the number of viral proteins that target a single host protein and found that the majority of human host proteins are targeted by a single viral protein (1.4±0.7, Fig. 3C). We analogously counted the number of viral proteins that HDF proteins indirectly interact with. In comparison to directly targeted host proteins, the corresponding mean value significantly shifted to higher numbers of viral proteins that indirectly interact with HDF proteins (4.6±3.0), a distribution that is significantly different (Student's t-test, P<10^−36^).

As for pathway specific aspects, we connected human host proteins if they significantly co-appeared in pathways utilizing a Fisher exact test (P<0.01). In a subsequent step, we constructed another network, connecting human host proteins if they significantly shared directly interacting viral proteins (P<0.01). Comparing the number of viral proteins and pathways that are shared by the underlying protein links we observed a strong and significant correlation (Fig. 3D; Pearson's r = 0.47, P<0.01). Such a result suggests that indeed the combinatorial ways the virus interacts with directly targeted proteins reflect the patterns of involvement in certain pathways. Similarly, we connected HDF proteins that significantly share indirectly interacting viral proteins (P<0.01) and found a similar, yet weaker correlation between pathway involvement and indirectly targeting viral proteins (inset, Fig. 3D; r = 0.26, P<0.1).

**Figure 4:**
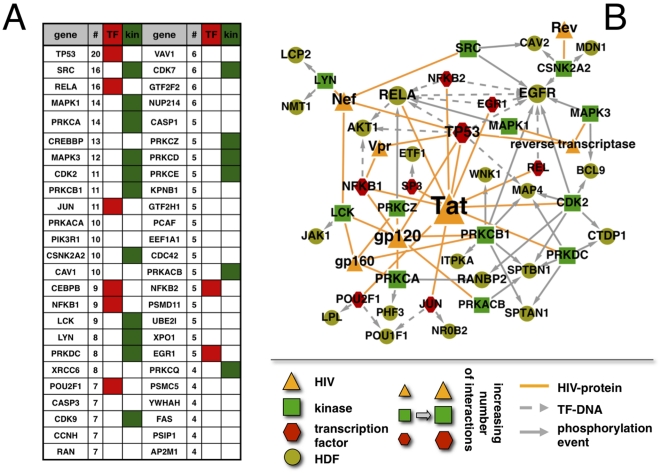
Combinations of viral proteins and map of direct and indirect interactions. In (**A**) we ranked targeted proteins according to the corresponding number of interactions with HIV dependency factor proteins (HDF). Showing the 50 most connected proteins we observed that transcription factors and kinases are enriched at the top of the list. In (**B**), we show a network of transcription factors and kinases that are attacked by viral proteins as well as their interactions with HDFs. Specifically, we observed that Tat largely interacts with prominent transcription factors, such as TP53 and NFKB1 and kinases, such as SRC and CDK2 which control important HDFs such as EGFR and RELA.

According to our definition, indirect interactions with a HDF are facilitated through interacting host proteins that are directly targeted by a viral protein. In Fig. 4A and Table S3, we ranked targeted proteins according to the corresponding number of interactions with a HDF, allowing us to observe that transcription factors and kinases such as TP53, RELA or SRC are enriched at the top of the list. Such an observation suggests that the process of seizing control of the host cell goes through well established interaction paths. Utilizing all transcription factors and kinases that facilitate an indirect interaction of viral proteins and HDFs, we show a network of such indirect and direct interactions in Fig. 4B. Specifically, we observed that Tat interacts with prominent transcription factors, including TP53 and NFKB1, as well as kinases, such as SRC and CDK2, which control important HDF proteins such as EGFR and RELA. These observations suggested that these important functional proteins are not only direct targets of the virus but also might serve as a further gateway to the control of downstream factors such as HDFs.

## Discussion

HIV-1 invokes intricate processes with a remarkably low number of proteins to take control of the human host cell. Compensating for its low number of proteins, combinations of pathogen proteins give the virus greater access to a broader set of human proteins. In particular, the subtle structure of the human interactome reveals sites that are not only topologically important, but also are targeted by HIV-1 in both direct and indirect ways. Specifically, rich clubs, protein assemblies that are strongly intertwined among each other, provide proteomic sites that are largely targeted by the virus. Although no direct interaction targets, HIV dependent factor proteins also provide such a proteomic characteristics that are indirectly exploited by the virus.

Since such proteins are at the intersection of numerous pathways, a large degree of interaction allows the virus to reach into many different functional processes. Subsets of viral proteins reach into the host network to ensure the largest, but focused diversity. Specifically, the use of direct and indirect targets in pathways leads to a cohesive pathway-dependent combination of targeted and HDF proteins. Since we found a strong correlation between the number of shared targeting viral proteins and pathways the underlying host proteins co-appear in, the virus potentially tailored its surface to attack the host cell along well established functional pathways. Recalling that targeted pathways harbor HDF proteins as well, we found a similar yet weaker trend for HDF proteins, suggesting that HDFs may act as downstream mediators of molecular viral information in the underlying pathways.

Utilizing HDFs in an indirect way the virus establishes its control over the host cell, indicating the particular systemic role of proteins that are not directly involved in physical host-pathogen interactions. Such proteins at the interface between the virus and the host are kinases and transcription factors. Such proteins are important mediators of molecular information that allow the virus to effectively utilize them as a gateway to interfere indirectly with a variety of different protein to take control of the human host and ensure the virus' survival. Therefore, untangling the intricate web of indirect and direct interactions is of utmost importance for a thorough understanding of the virus pathogenesis. In the light of these observations, transcription factors and kinases that provide access to proteins in an indirect way seem to be the key players in the subtle molecular strategies a virus employs in order to intercalate a host cell.

Observations that HDF proteins are enriched in rich-clubs, co-appear in many pathways and are largely linked to targeted proteins such as kinases and transcription factors might help to uncover virus dependent factors in systems where information about the interaction interface between a pathogen and a host cell is available. Assuming that the viral take-over of a host cell generally follows similar patterns [18] our results might be tapped to design computational approaches that allow us to predict virus dependable factor proteins in other host pathogen systems. Obviously, the computational prediction of viral dependent proteins offers an efficient and economical way to produce testable hypotheses that can be experimentally investigated further. In addition, the analysis of the entanglement of directly targeted and indirectly interacting proteins may uncover molecular and functional Achilles heels that could be used to systematically hamper viruses [23]. Consequently, defining the web of well defined direct and indirect host-pathogen interactions offers the opportunity to consider viral systems as naturally perturbed biological systems that can be utilized to identify and disentangle relevant pathways in different cellular contexts, ultimately allowing us to eradicate other pathogen driven diseases that plague human kind.

## Materials and Methods

### Human HIV Protein Interactions

We utilized a compilation of 702 experimentally obtained protein interactions between the human host and HIV-1, accounting for interactions that have been found in vital cells in the human immune system such as helper T cells, macrophages and dendritic cells [24].

### Protein Interaction and Pathway Data

Collecting pairwise protein interactions in *H. sapiens* from public databases [12], [25], [26] we obtained a network of 9,888 proteins embedded in 69,194 physical interactions.

As a reliable source of experimentally confirmed protein-DNA interactions, we used 6,669 interactions between 2,822 transcription factors and structural genes from the TRED database [27]. As for phosphorylation events between kinases and other proteins we found 5,462 interactions between 1,707 human proteins utilizing networKIN [28], [29] and phosphoELM database [30]. As a source of reliable human protein pathway information we utilized 913 annotated pathways from the Pathway Interaction Database [22].

### Rich-Club Coefficient

The so-called rich-club phenomenon is quantitatively defined by the rich-club coefficient 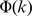 [20]. Denoting by 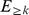 the number of edges among the nodes 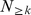 which have at least *k* interaction partners, the rich-club coefficient is expressed as 
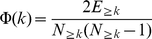
, where 
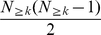
 represents the maximally possible number of edges among 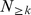 nodes. An appropriate choice for normalizing the rich-club coefficient is provided by the ratio 
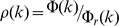
, where 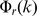 is the rich-club coefficient of a random network with the same degree distribution *P(k)*. In order to have a reasonably large ensemble, we repeated the randomization process 10,000 times. Binning nodes according to their degrees *k* we obtained a degree dependent mean value of the rich-club coefficient by averaging over all *ρ*'s in each bin. A ratio larger than one, *ρ*<1, is the actual evidence for the presence of a rich-club phenomenon, an increase in the interconnectivity of large degree nodes compared to the random case. This process is well displayed by the presence of an oligarchy of highly interacting nodes that are well connected among each other. A ratio *ρ*<1 points to a lack of interconnectivity among large degree nodes that are separated in distinguishable modules.

### Enrichment

Each rich club where each protein has at least *k* interactions *N_≥k_* is represented as a subset of all proteins *N* in the underlying network, 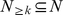. In order to obtain an estimate if proteins with a feature *a* are overrepresented in a rich-club, we calculated the corresponding fraction 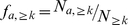 in the underlying rich club *N_≥k_*. As a null hypothesis, we assumed that the feature *a* is randomly distributed among human proteins. Determining the randomized fraction of such proteins 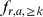, we defined 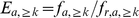 as the enrichment of proteins that have feature *a* in a rich club. Averaging *E* over 10,000 randomizations rich clubs are enriched with feature *a* if *E>1* and *vice versa*.

### Pathway Participation Coefficient

For each protein that is part of at least one pathway, we defined the pathway participation coefficient of a protein *i*, as 
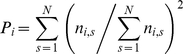
 where 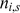 is the number of links protein *i* has to proteins in pathway *s* out of all *N* pathways. If a protein predominantly interacts with partners that are members of the same pathway, *P* tends to *1* while the opposite holds if the interaction partners are distributed among many different pathways.

### Significance of Attacked Pathways

Determining the significance of pathways that are enriched with proteins expressed in a human T-cell, we formed a 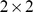 contingency table by determining α expressed proteins and the remainder of β proteins in a given pathway. While γ is the number of expressed proteins and δ is the number of remaining proteins in all the other pathways we calculated the probability of obtaining any such set of values randomly by 
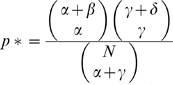
, where 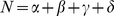. In order to investigate the two tails of the underlying distribution we constructed all possible contingency tables by keeping the sum of rows and columns constant. The P-value to reject the null hypothesis being the independence of rows and columns in the contingency table is the sum of the probabilities 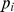, of all contingency tables *i* where 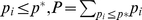 [31].

### Significance of Links between Proteins

We applied a hypergeometric distribution to model the probability of obtaining a number of shared features of proteins *v* and *w* at or above the observed number by chance. Considering a total of *T* proteins, we defined the significance that proteins *v* and *w* share similar features as
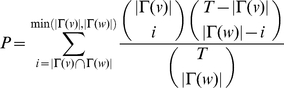
where *G(x)* represents the feature of protein *x*.

### Kernel Density Function

A simple way to analyze a series of values *x = x_1_, …, x_n_* would be a histogram. However, if the number of observations is low the significance of a histogram is rather limited. Therefore, we defined the kernel density approximation, a smoothing operation that allows the estimation of a putative probability density function of data points around a certain point *x* as 
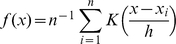
. *K*(*y*) is the kernel function, satisfying 
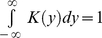
, and *h* is a smoothing parameter. In particular, we chose the Gaussian as kernel function 
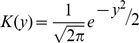
.

## Supporting Information

Table S1 List of 851 pathways that are enriched in the human host cell (P<0.05).
(0.49 MB XLS)

Table S2 79 HIV dependant factor proteins (HDF), their attacked proteins they are connected to and targeting viral proteins.
(0.03 MB XLS)

Table S3 Shows targeted genes that appear in shortest paths to HDFs (#: number of appearances in paths, TF: transcription factors, kin: kinases).
(0.03 MB XLS)
